# Bis(4-ammonio-4-methyl­pentan-2-one-κ*O*)dioxalato-κ^4^ *O* ^1^,*O* ^2^-copper(II)

**DOI:** 10.1107/S1600536808042396

**Published:** 2009-01-14

**Authors:** Zhiqin Ji, Shaopeng Wei, Wenjun Wu

**Affiliations:** a Institute of Pesticide Science, Northwest Agricultural & Forestry University, YangLing, Shaanxi 712100, People’s Republic of China

## Abstract

The title compound, [Cu(C_2_O_4_)_2_(C_6_H_14_NO)_2_], was synthesized by mixing diacetonamine hydrogen oxalate and copper sulfate in ethanol/water. The mol­ecule is centrosymmetric, so two pairs of equivalent ligands lie *trans* to each other. The Cu^II^ center, located on a position with 2/*m* site symmetry, is six-coordinated by four O atoms from two oxalate ligands at short distances and the carbonyl O atoms from the 4-amino-4-methyl­pentan-2-one ligands at longer distances. Mol­ecules are linked through inter­molecular N—H⋯O hydrogen bonds between the amino groups and carbonyl O atoms; no intra­molecular hydrogen bonds are formed.

## Related literature

For the preparation of diacetonamine, see: Haeseler (1925 ▶).
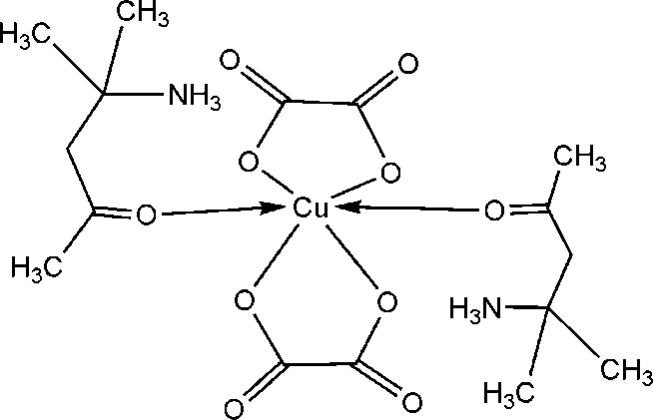


## Experimental

### 

#### Crystal data

[Cu(C_2_O_4_)_2_(C_6_H_14_NO)_2_]
*M* *_r_* = 471.94
Monoclinic, 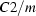

*a* = 13.639 (3) Å
*b* = 7.9749 (16) Å
*c* = 10.958 (2) Å
β = 113.27 (3)°
*V* = 1094.9 (4) Å^3^
*Z* = 2
Mo *K*α radiation
μ = 1.05 mm^−1^
*T* = 113 (2) K
0.16 × 0.14 × 0.14 mm

#### Data collection

Rigaku Saturn CCD area-detector diffractometer
Absorption correction: multi-scan (*CrystalClear*; Rigaku, 2005 ▶) *T*_min_ = 0.850, *T*_max_ = 0.867
4513 measured reflections
1394 independent reflections
1247 reflections with *I* > 2σ(*I*)
*R*_int_ = 0.033

#### Refinement

*R*[*F* ^2^ > 2σ(*F* ^2^)] = 0.026
*wR*(*F* ^2^) = 0.075
*S* = 1.11
1394 reflections
83 parameters
H atoms treated by a mixture of independent and constrained refinement
Δρ_max_ = 0.31 e Å^−3^
Δρ_min_ = −0.45 e Å^−3^

### 

Data collection: *CrystalClear* (Rigaku, 2005 ▶); cell refinement: *CrystalClear*; data reduction: *CrystalClear*; program(s) used to solve structure: *SHELXS97* (Sheldrick, 2008 ▶); program(s) used to refine structure: *SHELXL97* (Sheldrick, 2008 ▶); molecular graphics: *SHELXTL* (Sheldrick, 2008 ▶); software used to prepare material for publication: *CrystalStructure* (Rigaku, 2005 ▶).

## Supplementary Material

Crystal structure: contains datablocks I, global. DOI: 10.1107/S1600536808042396/jh2070sup1.cif

Structure factors: contains datablocks I. DOI: 10.1107/S1600536808042396/jh2070Isup2.hkl

Additional supplementary materials:  crystallographic information; 3D view; checkCIF report

## Figures and Tables

**Table:** 

Cu1—O1	1.9383 (11)
Cu1—O3	2.663 (2)

**Table:** 

O1—Cu1—O1^i^	179.999 (2)
O1—Cu1—O1^ii^	94.90 (6)
O1^i^—Cu1—O1^ii^	85.10 (6)

Symmetry codes: (i) 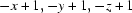; (ii) 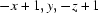.

**Table 2:** Hydrogen-bond geometry (Å, °)

*D*—H⋯*A*	*D*—H	H⋯*A*	*D*⋯*A*	*D*—H⋯*A*
N1—H1*A*⋯O2^iii^	0.86 (3)	2.23 (2)	2.950 (2)	141.8 (5)
N1—H1*A*⋯O2^iv^	0.86 (3)	2.23 (2)	2.950 (2)	141.8 (5)
N1—H1*B*⋯O2^v^	0.883 (18)	2.014 (19)	2.8651 (14)	161.5 (16)

Symmetry codes: (iii) 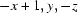; (iv) 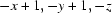; (v) 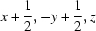.

**Fig. 1.**
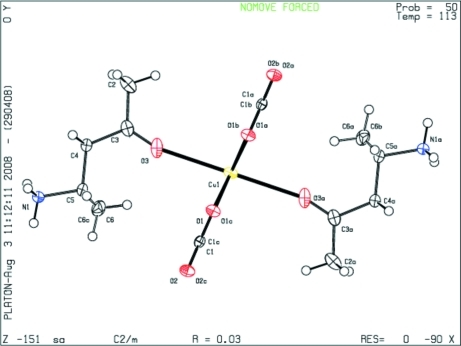
Molecular structure of the title compound, showing the coordination geometry. Displacement ellipsoids are drawn at the 50% probability level.

**Fig. 2.**
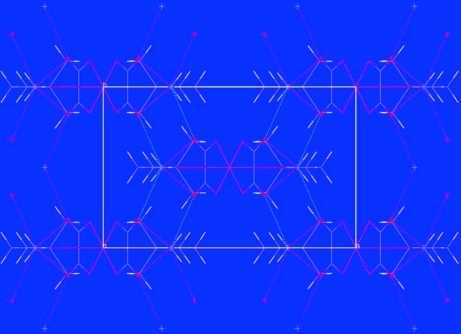
Packing diagram viewed down the c-axis,showing hydrogen bonds.

## supplementary crystallographic information

### Comment

In the screening of novel antibiotics, diacetonamine was obtained in the procedure of isolating active ingredients by the silica gel chromatography. Diacetonamine exhibites moderatly antimicrobial activities against many species of plant-pathogenic fungus. To enhance the bio-activity, a complex was designed and prepared by the mixture of diacetonamine hydrogen oxalate and copper sulfate. Compared with diacetonamine, the antimicrobial activities of copper complex was increased dramaticaly. Diacetonamine could be prepared from a mixture of mesityl oxide with aqueous ammonia or liquid ammonia(Haeseler, 1925). In this paper, [Cu(C_6_H_13_NO)_2_ (C_2_H_2_O_4_)_2_] was synthesized by the reaction of CuSO_4_.5H_2_O and diacetonamine hydrogen oxalate in ethanol/water and the structure of the resulting complex is presented herein.

### Experimental

Diacetonamine hydrogen oxalate(0.6 mmol 123 mg) was dissolved in ethnaol/water (2/1,volume ratio, 10 ml) and the solution was heated to boiling. Copper sulfate(0.3 mmol 75 mg) was dissolved in deionized water(10 ml), and was added dropwise to the solution and stirred for 10 minutes. The mother liquid was placed at room temperature, and single crystals were obtained on standing.

### Crystal data

**Table:**

[Cu(C_2_O_4_)_2_(C_6_H_14_NO)_2_]	*F*(000) = 494
*M**_r_* = 471.94	*D*_x_ = 1.432 Mg m^−^^3^
Monoclinic, *C*2/*m*	Mo *K*α radiation, λ = 0.71073 Å
*a* = 13.639 (3) Å	Cell parameters from 2107 reflections
*b* = 7.9749 (16) Å	θ = 3.3–27.9°
*c* = 10.958 (2) Å	µ = 1.05 mm^−^^1^
β = 113.27 (3)°	*T* = 113 K
*V* = 1094.9 (4) Å^3^	Prism, colorless
*Z* = 2	0.16 × 0.14 × 0.14 mm

### Data collection

**Table:**

Rigaku Saturn CCD area-detector diffractometer	1394 independent reflections
Radiation source: rotating anode	1247 reflections with *I* > 2σ(*I*)
confocal	*R*_int_ = 0.033
Detector resolution: 7.31 pixels mm^-1^	θ_max_ = 27.9°, θ_min_ = 3.3°
ω and φ scans	*h* = −17→17
Absorption correction: multi-scan (*CrystalClear*; Rigaku, 2005)	*k* = −10→9
*T*_min_ = 0.850, *T*_max_ = 0.867	*l* = −12→14
4513 measured reflections	

### Refinement

**Table:**

Refinement on *F*^2^	Primary atom site location: structure-invariant direct methods
Least-squares matrix: full	Secondary atom site location: difference Fourier map
*R*[*F*^2^ > 2σ(*F*^2^)] = 0.026	Hydrogen site location: inferred from neighbouring sites
*wR*(*F*^2^) = 0.075	H atoms treated by a mixture of independent and constrained refinement
*S* = 1.11	*w* = 1/[σ^2^(*F*_o_^2^) + (0.0374*P*)^2^ + 0.4743*P*] where *P* = (*F*_o_^2^ + 2*F*_c_^2^)/3
1394 reflections	(Δ/σ)_max_ = 0.001
83 parameters	Δρ_max_ = 0.31 e Å^−^^3^
0 restraints	Δρ_min_ = −0.45 e Å^−^^3^

### Special details

**Table:**

Geometry. All esds (except the esd in the dihedral angle between two l.s. planes) are estimated using the full covariance matrix. The cell esds are taken into account individually in the estimation of esds in distances, angles and torsion angles; correlations between esds in cell parameters are only used when they are defined by crystal symmetry. An approximate (isotropic) treatment of cell esds is used for estimating esds involving l.s. planes.
Refinement. Refinement of F^2^ against ALL reflections. The weighted R-factor wR and goodness of fit S are based on F^2^, conventional R-factors R are based on F, with F set to zero for negative F^2^. The threshold expression of F^2^ > 2sigma(F^2^) is used only for calculating R-factors(gt) etc. and is not relevant to the choice of reflections for refinement. R-factors based on F^2^ are statistically about twice as large as those based on F, and R- factors based on ALL data will be even larger.

### Fractional atomic coordinates and isotropic or equivalent isotropic displacement parameters (Å^2^)

**Table:**

	*x*	*y*	*z*	*U*_iso_*/*U*_eq_	
Cu1	0.5000	0.5000	0.5000	0.01576 (13)	
O1	0.44732 (8)	0.33564 (13)	0.35854 (10)	0.0177 (2)	
O2	0.36121 (9)	0.32743 (14)	0.13800 (10)	0.0200 (2)	
N1	0.76865 (14)	0.5000	0.11200 (17)	0.0153 (4)	
H1A	0.727 (2)	0.5000	0.029 (3)	0.023*	
H1B	0.8108 (14)	0.412 (2)	0.1262 (17)	0.023*	
O3	0.67707 (15)	0.5000	0.45267 (19)	0.0361 (4)	
C1	0.40342 (10)	0.40251 (18)	0.24483 (13)	0.0142 (3)	
C2	0.8619 (2)	0.5000	0.5942 (2)	0.0320 (6)	
H2A	0.8364	0.5000	0.6642	0.048*	
H2B	0.9045	0.4017	0.6013	0.048*	
C3	0.76883 (19)	0.5000	0.4625 (2)	0.0216 (5)	
C4	0.80035 (16)	0.5000	0.3451 (2)	0.0170 (4)	
H4A	0.8441	0.4031	0.3524	0.020*	
C5	0.71136 (16)	0.5000	0.2061 (2)	0.0168 (4)	
C6	0.64379 (13)	0.6585 (2)	0.17734 (17)	0.0274 (4)	
H6A	0.5934	0.6585	0.0841	0.041*	
H6B	0.6903	0.7570	0.1944	0.041*	
H6C	0.6042	0.6620	0.2349	0.041*	

### Atomic displacement parameters (Å^2^)

**Table:**

	*U*^11^	*U*^22^	*U*^33^	*U*^12^	*U*^13^	*U*^23^
Cu1	0.0216 (2)	0.00972 (19)	0.01316 (19)	0.000	0.00386 (14)	0.000
O1	0.0239 (6)	0.0113 (5)	0.0155 (5)	−0.0008 (4)	0.0053 (4)	0.0002 (4)
O2	0.0245 (6)	0.0152 (6)	0.0166 (5)	−0.0027 (4)	0.0042 (4)	−0.0028 (4)
N1	0.0160 (9)	0.0143 (9)	0.0147 (8)	0.000	0.0052 (7)	0.000
O3	0.0348 (10)	0.0458 (12)	0.0400 (10)	0.000	0.0280 (9)	0.000
C1	0.0133 (7)	0.0114 (7)	0.0189 (7)	−0.0013 (5)	0.0074 (6)	−0.0009 (5)
C2	0.0452 (15)	0.0325 (14)	0.0208 (11)	0.000	0.0156 (11)	0.000
C3	0.0303 (12)	0.0149 (10)	0.0248 (11)	0.000	0.0165 (9)	0.000
C4	0.0168 (10)	0.0172 (10)	0.0189 (10)	0.000	0.0089 (8)	0.000
C5	0.0152 (10)	0.0172 (11)	0.0200 (10)	0.000	0.0092 (8)	0.000
C6	0.0222 (8)	0.0298 (10)	0.0326 (9)	0.0100 (7)	0.0133 (7)	0.0059 (7)

### Geometric parameters (Å, °)

**Table:**

Cu1—O1^i^	1.9383 (11)	C1—C1^i^	1.555 (3)
Cu1—O1	1.9383 (11)	C2—C3	1.499 (3)
Cu1—O1^ii^	1.9383 (11)	C2—H2A	0.9601
Cu1—O1^iii^	1.9383 (11)	C2—H2B	0.9600
Cu1—O3	2.663 (2)	C3—C4	1.508 (3)
Cu1—O3	2.663 (2)	C4—C5	1.528 (3)
O1—C1	1.2672 (17)	C4—H4A	0.9601
O2—C1	1.2361 (17)	C5—C6	1.522 (2)
N1—C5	1.520 (3)	C5—C6^i^	1.522 (2)
N1—H1A	0.86 (3)	C6—H6A	0.9800
N1—H1B	0.883 (18)	C6—H6B	0.9800
O3—C3	1.213 (3)	C6—H6C	0.9800
			
O1^i^—Cu1—O1	85.10 (6)	O3—C3—C4	123.8 (2)
O1^i^—Cu1—O1^ii^	94.90 (6)	C2—C3—C4	113.71 (19)
O1—Cu1—O1^ii^	179.999 (2)	C3—C4—C5	117.95 (18)
O1^i^—Cu1—O1^iii^	180	C3—C4—H4A	107.8
O1—Cu1—O1^iii^	94.90 (6)	C5—C4—H4A	107.8
O1^ii^—Cu1—O1^iii^	85.10 (6)	N1—C5—C6	106.94 (11)
C1—O1—Cu1	112.56 (10)	N1—C5—C6^i^	106.94 (11)
C5—N1—H1A	114.0 (17)	C6—C5—C6^i^	112.28 (19)
C5—N1—H1B	110.8 (11)	N1—C5—C4	104.95 (16)
H1A—N1—H1B	107.6 (14)	C6—C5—C4	112.57 (11)
O2—C1—O1	126.13 (14)	C6^i^—C5—C4	112.57 (11)
O2—C1—C1^i^	118.98 (8)	C5—C6—H6A	109.5
O1—C1—C1^i^	114.89 (8)	C5—C6—H6B	109.5
C3—C2—H2A	109.5	H6A—C6—H6B	109.5
C3—C2—H2B	109.5	C5—C6—H6C	109.5
H2A—C2—H2B	109.5	H6A—C6—H6C	109.5
O3—C3—C2	122.5 (2)	H6B—C6—H6C	109.5
			
O1^i^—Cu1—O1—C1	−1.03 (11)	O3—C3—C4—C5	0.0
O1^ii^—Cu1—O1—C1	−71 (11)	C2—C3—C4—C5	180.0
O1^iii^—Cu1—O1—C1	178.97 (11)	C3—C4—C5—N1	180.0
Cu1—O1—C1—O2	−178.41 (11)	C3—C4—C5—C6	64.06 (12)
Cu1—O1—C1—C1^i^	0.84 (9)	C3—C4—C5—C6^i^	−64.06 (12)

Symmetry codes: (i) *x*, −*y*+1, *z*; (ii) −*x*+1, −*y*+1, −*z*+1; (iii) −*x*+1, *y*, −*z*+1.

### Hydrogen-bond geometry (Å, °)

**Table:**

*D*—H···*A*	*D*—H	H···*A*	*D*···*A*	*D*—H···*A*
N1—H1A···O2^iv^	0.86 (3)	2.23 (2)	2.950 (2)	142 (1)
N1—H1A···O2^v^	0.86 (3)	2.23 (2)	2.950 (2)	142 (1)
N1—H1B···O2^vi^	0.883 (18)	2.014 (19)	2.8651 (14)	161.5 (16)

Symmetry codes: (iv) −*x*+1, *y*, −*z*; (v) −*x*+1, −*y*+1, −*z*; (vi) *x*+1/2, −*y*+1/2, *z*.

## References

[bb1] Haeseler (1925). *J. Am. Chem. Soc.* 47, 1195**y**

[bb2] Rigaku (2005). *CrystalStructure* and *CrystalClear* Rigaku Corporation, Tokyo, Japan.

[bb4] Sheldrick, G. M. (2008). *Acta Cryst.* A**64**, 112–122. 10.1107/S0108767307043930 18156677

